# Concomitant Proton Pump Inhibitor Use With Pembrolizumab Monotherapy vs Immune Checkpoint Inhibitor Plus Chemotherapy in Patients With Non−Small Cell Lung Cancer

**DOI:** 10.1001/jamanetworkopen.2023.22915

**Published:** 2023-07-11

**Authors:** Hayato Kawachi, Tadaaki Yamada, Motohiro Tamiya, Yoshiki Negi, Yasuhiro Goto, Akira Nakao, Shinsuke Shiotsu, Keiko Tanimura, Takayuki Takeda, Asuka Okada, Taishi Harada, Koji Date, Yusuke Chihara, Isao Hasegawa, Nobuyo Tamiya, Masaki Ishida, Yuki Katayama, Kenji Morimoto, Masahiro Iwasaku, Shinsaku Tokuda, Takashi Kijima, Koichi Takayama

**Affiliations:** 1 Department of Pulmonary Medicine, Graduate School of Medical Science, Kyoto Prefectural University of Medicine, Kyoto, Japan; 2 Department of Thoracic Oncology, Osaka International Cancer Institute, Osaka, Japan; 3 Department of Respiratory Medicine and Hematology, School of Medicine, Hyogo Medical University, Nishinomiya, Hyogo, Japan; 4 Department of Respiratory Medicine, Fujita Health University School of Medicine, Toyoake, Aichi, Japan; 5 Department of Respiratory Medicine, Fukuoka University Hospital, Nanakuma, Fukuoka, Japan; 6 Department of Respiratory Medicine, Japanese Red Cross Kyoto Daiichi Hospital, Kyoto, Japan; 7 Department of Respiratory Medicine, Japanese Red Cross Kyoto Daini Hospital, Kyoto, Japan; 8 Department of Respiratory Medicine, Saiseikai Suita Hospital, Suita, Osaka, Japan; 9 Department of Medical Oncology, Fukuchiyama City Hospital, Fukuchiyama, Kyoto, Japan; 10 Department of Pulmonary Medicine, Kyoto Chubu Medical Center, Nantan, Kyoto, Japan; 11 Department of Respiratory Medicine, Uji-Tokushukai Medical Center, Uji, Kyoto, Japan; 12 Department of Respiratory Medicine, Saiseikai Shigaken Hospital, Rittou, Shiga, Japan; 13 Department of Respiratory Medicine, Rakuwakai Otowa Hospital, Kyoto, Japan

## Abstract

**Importance:**

Immune checkpoint inhibitor (ICI) monotherapy with pembrolizumab and ICI plus chemotherapy have been approved as first-line treatments for non–small cell lung cancer (NSCLC) for patients with a programmed cell death ligand–1 (PD-L1) tumor proportion score (TPS) of 50% or more, but the choice between these 2 therapeutic options is unclear.

**Objective:**

To clarify the association of a history of concurrent medication use with treatment outcomes for ICIs with or without chemotherapy in patients with NSCLC with a high PD-L1 TPS and to determine whether these clinical histories are biomarkers for appropriate treatment selection.

**Design, Setting, and Participants:**

This retrospective, multicenter cohort study at 13 hospitals in Japan included patients with advanced NSCLC with a PD-L1 TPS of 50% or more who had received pembrolizumab ICI monotherapy or ICI plus chemotherapy as the initial treatment between March 2017 and December 2020. The median (IQR) follow-up duration was 18.5 (9.2-31.2) months. Data were analyzed from April 2022 through May 2023.

**Exposure:**

ICI monotherapy with pembrolizumab or ICI plus chemotherapy as first-line treatment.

**Main Outcomes and Measures:**

The primary analysis was the association of treatment outcomes with baseline patient characteristics, including concomitant drug history, after propensity score matching. Cox proportional hazard models were used to determine the associations of patient characteristics with survival outcomes. Logistic regression analysis was used to determine the association of concomitant medication history with treatment outcomes and other patient characteristics.

**Results:**

A total of 425 patients with NSCLC were enrolled in the study including 271 patients (median [range] age, 72 [43-90] years; 215 [79%] men) who were treated with pembrolizumab monotherapy as the first-line treatment and 154 patients (median [range] age, 69 [36-86] years; 121 [79%] men) who were treated with ICI plus chemotherapy as the first-line treatment. In multivariable analysis, a history of proton pump inhibitor (PPI) use was independently associated with shorter progression-free survival (PFS) in the pembrolizumab monotherapy group (hazard ratio [HR], 1.38; 95% CI, 1.00-1.91; *P* = .048), but not in the ICI plus chemotherapy group. In patients with a PPI history, both the median (IQR) PFS (19.3 [9.0 to not reached] months vs 5.7 [2.4 to 15.2] months; HR, 0.38; 95% CI, 0.20-0.72; *P* = .002) and the median (IQR) overall survival (not reached [9.0 months to not reached) vs 18.4 [10.5 to 50.0] months; HR, 0.43; 95% CI, 0.20-0.92; *P* = .03) were significantly longer in the ICI plus chemotherapy group than in the pembrolizumab monotherapy group. In patients without a history of PPI use, both the median (IQR) PFS (18.8 months [6.6 months to not reached] vs 10.6 months [2.7 months to not reached]; HR, 0.81; 95% CI, 0.56-1.17; *P* = .26) and the median (IQR) overall survival (not reached [12.6 months to not reached] vs 29.9 [13.3 to 54.3] months, HR, 0.75; 95% CI, 0.48-1.18; *P* = .21) did not differ between groups.

**Conclusions and Relevance:**

This cohort study found that a history of PPI use could be an important clinical factor in treatment decision-making for patients with NSCLC with a PD-L1 TPS of 50% or more.

## Key Points

**Question:**

Among patients with advanced non–small cell lung cancer with high programmed cell death ligand–1 expression, could concomitant medications serve as biomarkers for the appropriate treatment selection of immune checkpoint inhibitor (ICI) monotherapy or ICI plus chemotherapy?

**Findings:**

In this cohort study of 425 patients with advanced non–small cell lung cancer, a history of proton pump inhibitor (PPI) use was associated with a shorter progression-free survival. Among patients with a history of PPI use, progression-free survival and overall survival were significantly longer in the ICI plus chemotherapy group than in the ICI pembrolizumab monotherapy group.

**Meaning:**

These findings suggest that a history of concomitant PPI use may be an important clinical factor that should be considered when choosing an ICI treatment with or without chemotherapy.

## Teaser

This cohort study analyzes the association of proton pump inhibitor use with treatment outcomes among patients in Japan with non−small cell lung cancer receiving immune checkpoint inhibitor therapy with or without chemotherapy.

## Introduction

Immune checkpoint inhibitors (ICIs) are antibodies that target programmed cell death–1 (PD-1) or programmed cell death ligand–1 (PD-L1) and have recently revolutionized treatment for various cancer types, particularly non–small cell lung cancer (NSCLC).^1,2,3,4,5,6^ To determine ICI treatment outcomes more accurately, clinical biomarkers are essential. One such biomarker is the PD-L1 tumor proportion score (TPS), which is assessed by immunohistochemistry; it is associated with improved treatment outcomes and is commonly used in clinical practice.^7,8^ In several clinical trials, ICI monotherapy for patients with advanced NSCLC with a high PD-L1 TPS (ie, ≥50%) has demonstrated superior outcomes compared with chemotherapy and has been established as a standard first-line treatment option.^4,6^ Furthermore, long-term follow-up data from the KEYNOTE-024 trial^9^ reported a 5-year progression-free survival (PFS) rate of 13% and a 5-year overall survival (OS) rate of 32% in this population, suggesting that durable benefits of pembrolizumab monotherapy may be limited, even in patients with NSCLC with high PD-L1 TPS.

Several phase 3 clinical trials^7,8,10,11,12^ previously reported that ICI plus chemotherapy exhibited superior outcomes compared with standard chemotherapy for patients with advanced NSCLC without oncogenic driver alterations, regardless of PD-L1 TPS. Furthermore, similar to ICI monotherapy, long-term follow-up analysis in some clinical trials^13,14^ has demonstrated that the outcomes of ICI plus chemotherapy are also associated with PD-L1 expression. Moreover, with the immunogenic effects of cytotoxic chemotherapy, modulation of the immune response through ICIs is considered to be enhanced with ICI plus chemotherapy.^15^ Nevertheless, in analyses of empirical data and pooled randomized clinical trial data, the long-term benefit of combining chemotherapy with ICIs remains unclear.^16,17,18^ Additionally, the potential increase in treatment-related toxic effects when adding chemotherapy to ICIs is a concern.^7,8,10,11,12^ Consequently, the optimal treatment for patients with NSCLC with a PD-L1 TPS of 50% or more (ie, ICI with vs without chemotherapy) is controversial. Therefore, additional factors need to be explored for optimal treatment selection for patients in clinical settings.

Previous reports^19,20,21,22,23^ showed that the concomitant use of drugs affecting gut microbiota was associated with positive ICI monotherapy outcomes. Among these drugs, histories of proton pump inhibitor (PPI) use and antibiotic use were associated with poorer outcomes in patients with NSCLC who were treated with ICI monotherapy but not in those treated with cytotoxic chemotherapy.^19,20,21,22,23^ We thus aimed to clarify the association of concurrent medication history with treatment outcomes in patients with NSCLC with a high PD-L1 TPS treated with ICI with or without chemotherapy, and to determine whether these clinical histories could serve as biomarkers for appropriate treatment selection.

## Methods

### Study Design and Patients

This retrospective multicenter cohort study was conducted in accordance with the Declaration of Helsinki^24^ and the World Health Organization’s Guidelines for Good Clinical Practice.^25^ This study was approved by the ethics review board of the Kyoto Prefectural University of Medicine and was conducted with consent from the ethics review board of each hospital involved in the study. Informed patient consent was not required given the retrospective nature of the study. This study was conducted at 13 institutions in Japan. Patients with consecutive advanced NSCLC (stage IV, including postoperative recurrence according to the American Joint Committee on Cancer Staging Manual^26^, version 8) who had received pembrolizumab monotherapy or ICI plus chemotherapy as the initial treatment between March 2017 and December 2020 were included.^27^ Patients with sensitizing epidermal growth factor receptor variant, anaplastic lymphoma kinase fusion, or c-ros oncogene 1 (ROS1) rearrangement were excluded. Patients with NSCLC recurrence were eligible if the recurrence occurred more than 24 weeks after the last perioperative chemotherapy administration. Patients who received a combination of uracil and tegafur as perioperative chemotherapy were eligible, regardless of the time elapsed between the last treatment and recurrence.

Clinical data relevant to first-line treatment initiation, including concomitant medications, were collected from electronic medical records. Elderly patients were defined as those aged 75 years or older, according to the Japanese Lung Cancer Society Guideline.^28^ PD-L1 TPS in tumor cells was analyzed using PD-L1 immunohistochemistry with a 22C3 pharmDx antibody (clone 22C3; Dako North America, Inc). Information on concomitant medications included the following: corticosteroids (dose ≥10 mg per day of a prednisone equivalent, with a minimum 24 hours of dosing) within the 30 days before first-line treatment initiation (excluding first-line treatment premedications); systemic antibiotics within 30 days before first-line treatment initiation; and PPI use at first-line treatment initiation.^19,20,29,30,31,32,33^

### Assessment of Outcomes

First, we investigated the association of patient-related factors, including concomitant medication history, with PFS in patients receiving pembrolizumab monotherapy or ICI plus chemotherapy. Then, we identified the patient-related factors significantly associated with pembrolizumab monotherapy or ICI plus chemotherapy outcomes and compared the outcomes between patients with and without these factors who received pembrolizumab monotherapy or ICI plus chemotherapy. When comparing the treatment outcomes between pembrolizumab monotherapy and ICI plus chemotherapy groups, we adjusted for significant differences in the baseline characteristics of patients using propensity score matching (PSM) for the following variables: age, sex, smoking status, Eastern Cooperative Oncology Group performance status (ECOG PS), histologic profile, PD-L1 TPS, cancer stage, liver metastasis, and brain metastasis. Nearest neighbor matching was performed at a ratio of 1:1 without replacement and a caliper of 0.2. Treatment response was evaluated using the Response Evaluation Criteria in Solid Tumors, version 1.1.^34^ PFS was measured from the start of first-line treatment until the first instance of lung cancer progression or any-cause death. OS was measured from the start of first-line treatment until any-cause death. The data cutoff date for follow-up was September 30, 2021. The median (IQR) follow-up duration was 18.5 (9.2-31.2) months.

### Statistical Analysis

Age was analyzed using the Wilcoxon rank-sum test. Dichotomous variables were analyzed using the χ^2^ test or Fisher exact test, as appropriate. Survival outcomes were estimated using the Kaplan-Meier method and were compared using the log-rank test. Cox proportional hazard models were fitted to determine the associations of patient characteristics with survival outcomes. Logistic regression analysis was used to determine the association of concomitant medication history with treatment outcome and other patient characteristics. The results are expressed as odds ratios (ORs) or hazard ratios (HRs) with 95% CIs, as appropriate. All analyses were performed using JMP statistical software version 14 (SAS Institute). Statistical significance was defined as 2-tailed *P* < .05. Statistical analyses were performed from April 2022 to May 2023.

## Results

### Patient Characteristics

A total of 425 patients with NSCLC were enrolled in the study. A total of 271 patients (median [range] age, 72 [43-90] years; 215 [79%] men) were treated with pembrolizumab monotherapy as the first-line treatment, and 154 patients (median [range] age, 69 [36-86] years; 121 [79%] men) were treated with ICI plus chemotherapy as the first-line treatment. The baseline characteristics of all patients are summarized in eTable 1 in Supplement 1. Compared with the ICI plus chemotherapy group, the pembrolizumab monotherapy group had significantly higher proportions of older patients (72 years vs 69 years ), patients aged 75 years or older (106 patients [39%] vs 20 patients [13%]; χ^2^ = 32.1; *P* < .001), patients with poor ECOG PS (56 patients [21%] vs 15 patients [10%]; χ^2^ = 8.4; *P* = .004), patients administered PPIs (95 patients [35%] vs 39 patients [25%]; χ^2^ = 4.3; *P* = .04), and patients administered antibiotics (52 patients [19%] vs 18 patients [12%]; χ^2^ = 4.0; *P* = .045). PD-L1 TPS and cancer stage (IVA, IVB, and recurrence) differed significantly between the 2 groups. After PSM weighting, 131 patients matched patients in each group, with no significant differences in baseline characteristics between the 2 matched groups (Table 1).

**Table 1.** Patient Characteristics Adjusted by Propensity Score Matching

Characteristics	Patients, No. (%) (N = 262)		*P* value
	Pembrolizumab monotherapy group (n = 131)	ICI plus chemotherapy group (n = 131)	
Age, y			
Median (range)	69 (45-84)	69 (36-86)	.19
<75	22 (17)	20 (15)	.74
≥75	109 (83)	111 (85)	
Sex			
Male	109 (83)	107 (82)	.75
Female	22 (17)	24 (18)	
Smoking status			
Never smoked	13 (10)	15 (11)	.69
Current or former smoker	118 (90)	116 (89)	
Eastern Cooperative Oncology Group performance status			
0-1	120 (92)	116 (89)	.41
2-4	11 (8)	15 (11)	
Histologic profile			
Squamous cell carcinoma	34 (26)	36 (27)	.93
Adenocarcinoma	78 (60)	75 (57)	
Other	19 (15)	20 (15)	
Programmed cell death ligand 1 tumor proportion score, %			
50-74	43 (33)	51 (39)	.59
75-89	34 (26)	31 (24)	
90-100	54 (41)	49 (37)	
Stage			
IVA	47 (36)	44 (34)	.82
IVB	64 (49)	69 (53)	
Recurrence	20 (15)	18 (14)	
Proton pump inhibitor			
Administered	39 (30)	31 (24)	.26
Not administered	92 (70)	100 (76)	
Antibiotics			
Administered	26 (20)	15 (11)	.06
Not administered	105 (80)	116 (89)	
Steroids			
Administered	3 (2)	6 (5)	.31
Not administered	128 (98)	125 (95)	
Body mass index^a^			
<20	38 (29)	39 (30)	.89
≥20	93 (71)	92 (70)	
Liver metastasis	16 (12)	20 (15)	.47
Brain metastasis	21 (16)	22 (17)	.87
Treatment regimen			
Pembrolizumab	131 (100)		NA
Carboplatin, paclitaxel, and pembrolizumab	NA	1 (1)	NA
Carboplatin, nanoparticle albumin-bound paclitaxel, and pembrolizumab	NA	45 (34)	NA
Carboplatin, pemetrexed, and pembrolizumab	NA	33 (25)	NA
Cisplatin, pemetrexed, and pembrolizumab	NA	23 (18)	NA
Carboplatin, pemetrexed, and atezolizumab	NA	1 (1)	NA
Carboplatin, paclitaxel, and atezolizumab	NA	1 (1)	NA
Carboplatin, paclitaxel, bevacizumab, and atezolizumab	NA	17 (13)	NA
Carboplatin, nanoparticle albumin-bound paclitaxel, and atezolizumab	NA	10 (8)	NA

Abbreviations: ICI, immune checkpoint inhibitor; NA; not applicable.

^a^ Body mass index is calculated as weight in kilograms divided by height in meters squared.

### Treatment Outcomes in All Patients

Overall, the objective response rate (73.4% vs 55.3%; χ^2^ = 11.8; *P* < .001) and disease control rate (92.1% vs 76.0%; χ^2^ = 15.0; *P* < .001) were significantly higher in the ICI plus chemotherapy than in the pembrolizumab group (eFigure 1A in Supplement 1). The median (IQR) PFS in the ICI plus chemotherapy group was longer than that in the pembrolizumab monotherapy group (17.3 months [6.4 months to not reached] vs 10.6 [3.4 to 52.9] months), but the HR was not statistically significant (HR, 0.77; 95% CI, 0.59-1.00; *P* = .05) (eFigure 1B in Supplement 1). The median (IQR) OS was significantly longer in the ICI plus chemotherapy group than in the pembrolizumab monotherapy group (not reached [13.5 months to not reached] vs 25.6 [10.5 to 55.6] months; HR, 0.69; 95% CI, 0.50-0.95; *P* = .03) (eFigure 1C in Supplement 1).

**Figure 1.**
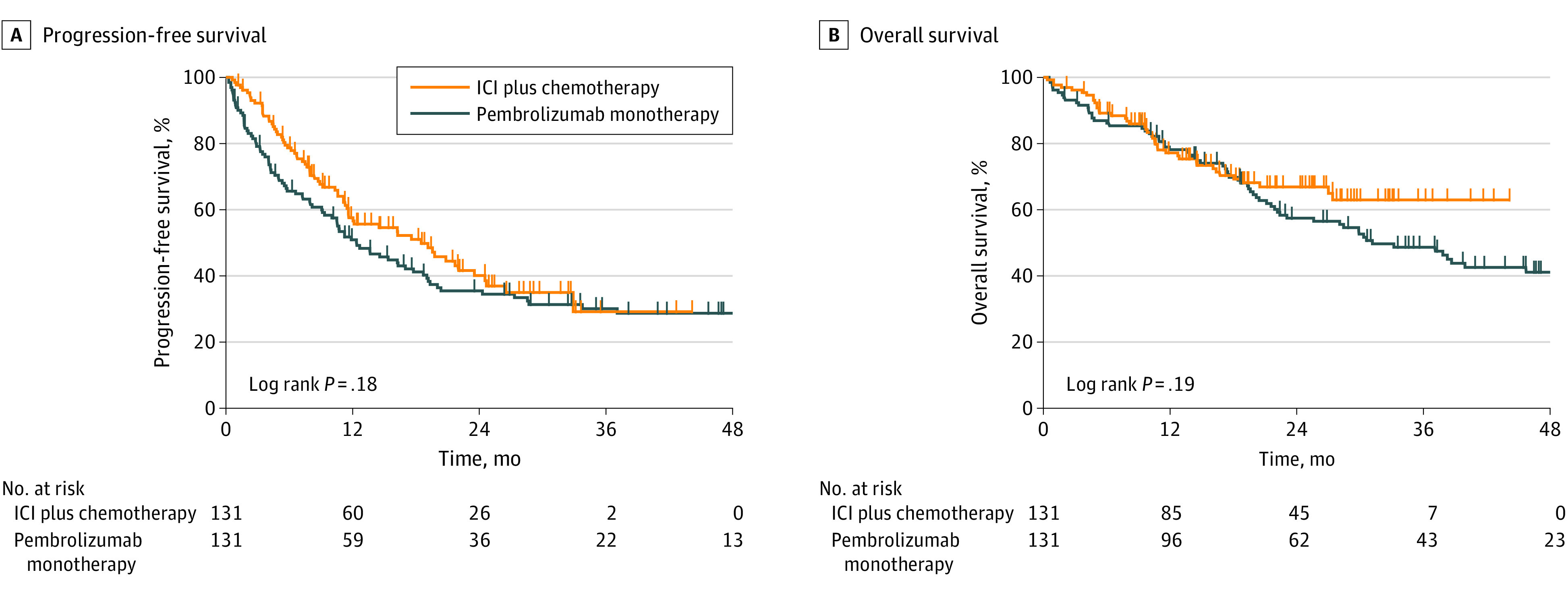
Treatment Outcome Adjusted by Propensity Score Matching in All Patients The figure shows Kaplan-Meier survival curves for progression-free survival (A) and overall survival (B) for patients receiving immune checkpoint inhibitor (ICI) monotherapy (pembrolizumab) and ICI plus chemotherapy (N = 262).

Analysis of the treatment outcomes after PSM showed that the objective response rate (76.7% vs 59.2%; χ^2^ = 7.7; *P* = .005) and disease control rate (94.0% vs 77.7%; χ^2^ = 12.3; *P* < .001) were significantly higher in the ICI plus chemotherapy group than in the pembrolizumab monotherapy group (eFigure 2 in Supplement 1). The median (IQR) PFS was similar (18.2 months [7.1 months to not reached] vs 12.2 months [4.0 months to not reached]) between the ICI plus chemotherapy and pembrolizumab monotherapy groups (HR, 0.80; 95% CI, 0.58-1.11; *P* = .18) (Figure 1A). The median (IQR) OS (not reached [14.3 months to not reached] vs 30.7 months [14.4 months to not reached]) was also similar between the 2 groups (HR, 0.77; 95% CI, 0.51-1.14; *P* = .19) (Figure 1B).

### Treatment Outcomes According to Patient Characteristics in Each Treatment Group

We next analyzed the association of PFS with patient characteristics in each group. The results for the pembrolizumab monotherapy group are shown in Table 2. Univariate analysis using Cox proportional hazards models indicated that a PD-L1 TPS of 50% to 89% (HR, 1.56; 95% CI, 1.14-2.13; *P* = .005), history of PPI administration (HR, 1.43; 95% CI, 1.06-1.94; *P* = .02), and history of antibiotic administration (HR, 1.56, 95% CI, 1.10-2.21; *P* = .01) were significantly associated with PFS. According to the multivariable Cox proportional hazards regression model, PD-L1 TPS of 50% to 89% (HR, 1.65; 95% CI, 1.18-2.31; *P* = .003) and a PPI administration history (HR, 1.38; 95% CI, 1.00-1.91; *P* = .048) were independently associated with a shorter PFS.

**Table 2.** Analyses of Progression-Free Survival in Patients Administered Immune Checkpoint Inhibitor Monotherapy (Pembrolizumab)

Characteristics	Patients, No. (%) (N = 271)	Progression-free survival, median (IQR) mo	Univariate analysis		Multivariable analysis	
			HR (95% CI)	*P* value	HR (95% CI)	*P* value
Age, y						
<75	165 (61)	10.8 (3.2 to 60.9)	0.92 (0.68-1.25)	.61	0.92 (0.68-1.27)	.63
≥75	106 (39)	8.9 (3.7 to 52.9)	1 [Reference]	NA	1 [Reference]	NA
Sex						
Female	56 (21)	10.6 (2.8 to 27.0)	1.11 (0.77-1.59)	.57	1.14 (0.74-1.75)	.56
Male	215 (79)	10.6 (3.4 to 52.9)	1 [Reference]	NA	1 [Reference]	NA
Smoking status						
Current or former smoker	243 (90)	10.6 (3.2 to 52.9)	0.82 (0.51-1.31)	.40	0.80 (0.47-1.37)	.42
Never smoked	28 (10)	7.2 (3.4 to 22.5)	1 [Reference]	NA	1 [Reference]	NA
Eastern Cooperative Oncology Group Performance Status						
0-1	215 (79)	12.5 (4.0 to 60.9)	0.55 (0.38-0.78)	<.001	0.68 (0.46-1.01)	.06
2-4	56 (21)	4.7 (1.7 to 16.5)	1 [Reference]	NA	1 [Reference]	NA
Histologic profile						
Squamous cell carcinoma	80 (30)	10.4 (2.7 to 28.2)	1.11 (0.80-1.53)	.53	1.11 (0.78-1.58)	.55
Nonsquamous cell carcinoma	191 (70)	11.1 (3.4 to 52.9)	1 [Reference]	NA	1 [Reference]	NA
Programmed cell death ligand 1 tumor proportion score, %						
50-89	167 (62)	6.8 (2.5 to 28.2)	1.56 (1.14-2.13)	.005	1.65 (1.18-2.31)	.003
≥90	104 (38)	16.8 (7.1 to not reached)	1 [Reference]	NA	1 [Reference]	NA
Stage						
IVA or IVB	212 (78)	9.9 (2.8 to 36.6)	1.28 (0.89-1.83)	.19	1.25 (0.85-1.83)	.25
Recurrence	59 (22)	16.1 (4.5 to 52.9)	1 [Reference]	NA	1 [Reference]	NA
Proton pump inhibitor						
Administered	95 (35)	7.5 (2.7 to 23.9)	1.43 (1.06-1.94)	.02	1.38 (1.00-1.91)	.048
Not administered	176 (65)	13.1 (4.0 to 60.9)	1 [Reference]	NA	1 [Reference]	NA
Antibiotics						
Administered	52 (19)	5.7 (1.7 to 18.9)	1.56 (1.10-2.21)	.01	1.28 (0.88-1.88)	.20
Not administered	219 (81)	11.1 (3.9 to 64.8)	1 [Reference]	NA	1 [Reference]	NA
Steroids						
Administered	14 (5)	4.6 (1.7 to 23.9)	1.74 (0.99-3.06)	.06	1.36 (0.72-2.60)	.34
Not administered	257 (95)	10.8 (3.4 to 52.9)	1 [Reference]	NA	1 [Reference]	NA
Body mass index^a^						
<20	80 (30)	7.4 (2.1 to 25.3)	1.36 (0.99-1.86)	.06	1.26 (0.89-1.77)	.19
≥20	191 (70)	12.2 (3.8 to 52.9)	1 [Reference]	NA	1 [Reference]	NA
Liver metastasis						
Yes	35 (13)	3.2 (0.9 to not reached)	1.51 (0.98-2.35)	.06	1.24 (0.77-1.99)	.37
No	236 (87)	11.1 (4.0 to 52.9)	1 [Reference]	NA	1 [Reference]	NA
Brain metastasis						
Yes	51 (19)	8.1 (2.4 to 35.4)	1.10 (0.76-1.58)	.62	0.87 (0.57-1.34)	.37
No	220 (81)	10.8 (3.5 to 52.9)	1 [Reference]	NA	1 [Reference]	NA

Abbreviations: HR, hazard ratio; NA, not applicable.

^a^ Body mass index is calculated as weight in kilograms divided by height in meters squared.

The association of PFS with patient characteristics in the ICI plus chemotherapy group is shown in Table 3. Univariate analysis using Cox proportional hazards models indicated that smoking history (HR, 0.42; 95% CI, 0.24-0.74; *P* = .002), ECOG PS (HR, 0.33, 95% CI, 0.18-0.61; *P* < .001), and liver metastasis (HR, 1.72; 95% CI, 1.02-2.90; *P* = .04) were significantly associated with PFS. According to the multivariable Cox proportional hazards regression model, smoking history (HR, 0.39; 95% CI, 0.19-0.80; *P* = .01) and ECOG PS (HR, 0.26; 95% CI, 0.13-0.50; *P* < .001) were independently associated with a shorter PFS. In contrast to the pembrolizumab monotherapy group, no history of concomitant drug use was significantly associated with PFS in the ICI plus chemotherapy group.

**Table 3.** Analyses of Progression-Free Survival in Patients Administered Immune Checkpoint Inhibitor Plus Chemotherapy

Characteristics	Patients, No. (%) (N = 154)	Progression-free survival, median (IQR) mo	Univariate analysis		Multivariable analysis	
			HR (95% CI)	*P* value	HR (95% CI)	*P* value
Age, y						
<75	134 (87)	18.2 (6.1 to not reached)	0.95 (0.50-1.78)	.86	0.80 (0.42-1.55)	.52
≥75	20 (13)	17.3 (7.4 to 26.1)	1 [Reference]	NA	1 [Reference]	NA
Sex						
Female	33 (21)	10.2 (7.1 to 23.2)	1.41 (0.85-2.34)	.19	0.96 (0.50-1.86)	.91
Male	121 (79)	19.3 (6.1 to not reached)	1 [Reference]	NA	1 [Reference]	NA
Smoking status						
Current or former smoker	133 (86)	19.5 (6.7 to not reached)	0.42 (0.24-0.74)	.002	0.39 (0.19-0.80)	.01
Never smoked	21 (14)	7.2 (4.5 to 11.4)	1 [Reference]	NA	1 [Reference]	NA
Eastern Cooperative Oncology Group Performance Status						
0-1	139 (90)	18.9 (7.4 to not reached)	0.33 (0.18-0.61)	<.001	0.26 (0.13-0.50)	<.001
2-4	15 (10)	4.6 (2.3 to 11.0)	1 [Reference]	NA	1 [Reference]	NA
Histologic profile						
Squamous cell carcinoma	40 (26)	11.0 (5.4 to not reached)	1.38 (0.85-2.22)	.19	1.59 (0.95-2.67)	.08
Nonsquamous cell carcinoma	114 (74)	19.3 (6.6 to not reached)	1 [Reference]	NA	1 [Reference]	NA
Programmed cell death ligand 1 tumor proportion score, %						
50-89	104 (68)	11.5 (6.1 to not reached)	1.56 (0.96-2.55)	.08	1.63 (0.97-2.73)	.06
≥90	50 (32)	24.2 (6.7 to 32.4)	1 [Reference]	NA	1 [Reference]	NA
Stage						
IVA or IVB	136 (88)	16.0 (5.5 to not reached)	1.35 (0.70-2.62)	.38	1.59 (0.78-3.26)	.20
Recurrence	18 (12)	21.7 (11.4 to not reached)	1 [Reference]	NA	1 [Reference]	NA
Proton pump inhibitor						
Administered	39 (25)	20.4 (9.0 to not reached)	0.73 (0.43-1.23)	.24	0.83 (0.48-1.45)	.52
Not administered	115 (75)	14.3 (6.1 to 32.4)	1 [Reference]	NA	1 [Reference]	NA
Antibiotics						
Administered	18 (12)	12.0 (5.2 to 20.4)	1.34 (0.69-2.61)	.39	1.19 (0.57-2.50)	.64
Not administered	136 (88)	17.3 (6.4 to not reached)	1 [Reference]	NA	1 [Reference]	NA
Steroids						
Administered	7 (5)	24.2 (4.0 to not reached)	0.67 (0.21-2.15)	.50	0.47 (0.14-1.64)	.24
Not administered	147 (95)	16.0 (6.4 to not reached)	1 [Reference]	NA	1 [Reference]	NA
Body mass index^a^						
<20	48 (31)	24.2 (5.2 to not reached)	1.14 (0.71-1.83)	.60	1.07 (0.63-1.83)	.79
≥20	106 (69)	17.3 (6.4 to not reached)	1 [Reference]	NA	1 [Reference]	NA
Liver metastasis						
Yes	23 (15)	11.0 (4.8 to 20.5)	1.72 (1.02-2.90)	.04	1.70 (0.97-3.00)	.07
No	131 (85)	19.3 (7.1 to not reached)	1 [Reference]	NA	1 [Reference]	NA
Brain metastasis						
Yes	28 (18)	11.4 (4.0 to not reached)	1.22 (0.69-2.13)	.49	1.60 (0.85-3.03)	.15
No	126 (82)	18.2 (7.1 to not reached)	1 [Reference]	NA	1 [Reference]	NA

Abbreviations: HR, hazard ratio; NA, not applicable.

^a^ Body mass index is calculated as weight in kilograms divided by height in meters squared.

### Treatment Outcomes in Patients With or Without PPI Use

**Figure 2.**
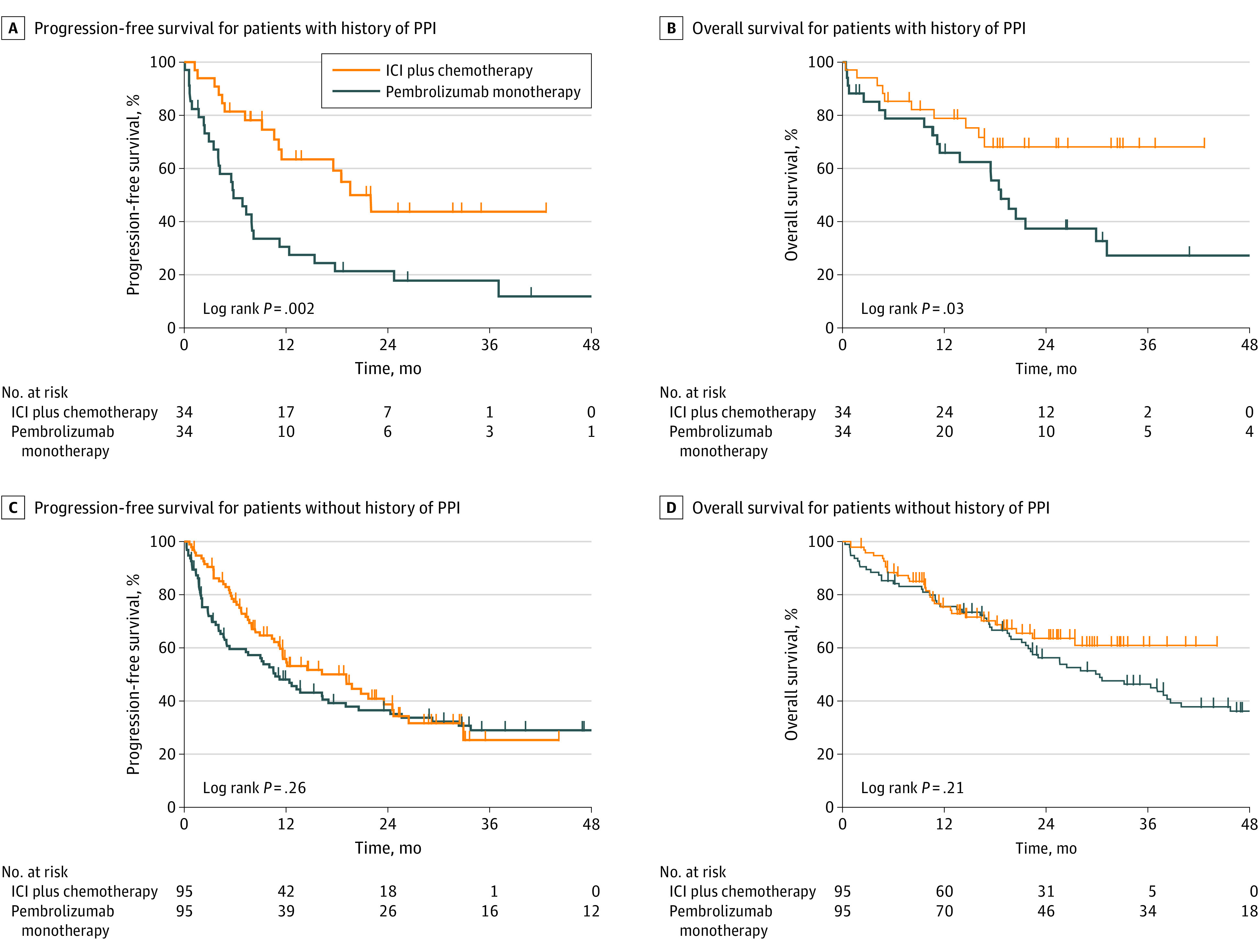
Treatment Outcome Adjusted by Propensity Scores According to History of Proton Pump Inhibitor (PPI) Use The figure shows Kaplan-Meier survival curves for progression-free survival (A) and overall survival (B) in patients receiving immune checkpoint inhibitor (ICI) monotherapy (pembrolizumab) and ICI plus chemotherapy who had a history of PPI use (n = 68) and the progression-free survival (C) and overall survival (D) in patients receiving ICI monotherapy (pembrolizumab) and ICI plus chemotherapy who did not have a history of PPI use (n = 190).

On the basis of the results of PFS according to concomitant drug use in each group, we compared the treatment outcomes between the pembrolizumab monotherapy group and ICI plus chemotherapy group in patients with or without a history of PPI use. After PSM weighting, 34 patients with and 95 patients without a history of PPI use were included in each group. There were no significant differences in baseline characteristics between the 2 groups (eTable 2 and eTable 3 in Supplement 1). In patients with a history of PPI use, both the median (IQR) PFS (19.3 months [9.0 months to not reached] vs 5.7 [2.4 to 15.2] months; HR, 0.38; 95% CI, 0.20-0.72; *P* = .002) (Figure 2A) and the median (IQR) OS (not reached [15.8 months to not reached] vs 18.4 [10.5 to 50.0] months; HR, 0.43; 95% CI, 0.20-0.92; *P* = .03) were significantly longer in the ICI plus chemotherapy group than in the pembrolizumab monotherapy group (Figure 2B). In contrast, in patients without a history of PPI use, both the median (IQR) PFS (18.8 months [6.6 months to not reached] vs 10.6 months [2.7 months to not reached]; HR, 0.81; 95% CI, 0.56 to 1.17; *P* = .26) (Figure 2C) and the median (IQR) OS (not reached [12.6 months to not reached] vs 29.9 [13.3 to 54.3] months; HR, 0.75; 95% CI, 0.48-1.18; *P* = .21) (Figure 2D) were similar between the ICI plus chemotherapy and pembrolizumab monotherapy groups.

### Patient Characteristics Associated With History of PPI Use

 We analyzed the patient factors associated with a history of PPI administration using logistic regression analysis. In the univariate analysis, patients with a history of PPI use were significantly more likely to be aged 75 years or older (OR, 1.77; 95% CI, 1.14-2.74; *P* = .01) and to have a smoking history (OR, 2.21; 95% CI, 1.04-4.71; *P* = .04), poor ECOG PS (OR, 2.03; 95% CI, 1.20-3.41; *P* = .008), a history of antibiotic administration (OR, 2.59; 95% CI, 1.53-4.36; *P* < .001), and a history of steroid administration (OR, 3.80; 95% CI, 1.54-9.41; *P* = .004). In multivariable logistic regression analyses, patients with a history of PPI use were significantly more likely to be aged 75 years or older (OR, 1.73; 95% CI, 1.10-2.75; *P* = .02) and to have a smoking history (OR, 2.72; 95% CI, 1.11-6.67; *P* = .03), antibiotic administration history (OR, 2.40; 95% CI, 1.37-4.21; *P* = .002), and steroid administration history (OR, 3.86; 95% CI, 1.37-10.93; *P* = .01), independent of other patient characteristics (eTable 4 in Supplement 1).

## Discussion

Both ICI monotherapy and ICI plus chemotherapy are standard treatments for patients with advanced NSCLC with a high PD-L1 TPS, and useful biomarkers are needed to guide the optimal treatment selection. In this cohort study, we showed that a history of PPI use was associated with a shorter PFS in patients with advanced NSCLC with a high PD-L1 TPS receiving pembrolizumab monotherapy but not in those receiving ICI plus chemotherapy. Furthermore, in patients with a history of PPI use, PFS and OS were significantly longer in the ICI plus chemotherapy than in the pembrolizumab monotherapy group. To the best of our knowledge, no previous study has demonstrated that a history of PPI use could be an important factor to consider in decision-making about using either ICI alone or ICI plus chemotherapy for patients with advanced NSCLC with high PD-L1 TPS in clinical settings.

PPIs are used to elevate the stomach’s pH by inhibiting stomach acid production. They are commonly used to manage gastrointestinal tract–related conditions, including hemorrhagic peptic ulcers, erosive esophagitis, and gastroesophageal reflux.^35,36,37^ However, PPI use is thought to compromise the protective barrier provided by stomach acid, allowing for the survival and colonization of the stomach by oropharyngeal and environmental microbiota. This colonization may extend into the small and large intestines, resulting in intestinal dysbiosis.^38^ PPI use has been associated with increases in the *Lactobacillales* order, particularly in the family *Streptococcaceae*, which may result from the downward movement of upper gastrointestinal tract commensals.^39^ Previous studies,^40,41,42,43^ including those from our study group, indicated that the gut microbiome composition is associated with the clinical benefits of ICI treatment in various types of cancer. On the basis of these findings, a history of PPI use is considered to be negatively associated with the therapeutic outcomes of ICI monotherapy, as previously reported.^19,20,21,22,23^

Similar to previous studies, our observations also showed that PPI administration might be associated with negative outcomes of ICI monotherapy in patients with NSCLC. Nevertheless, our research indicated that prior PPI use was not significantly associated with PFS in patients with NSCLC with high PD-L1 TPS receiving ICI plus chemotherapy. Although several studies^19,20,21,22,23^ have previously documented that prior PPI use was inversely associated with both PFS and OS of patients undergoing ICI monotherapy, studies exploring the clinical impact of PPIs on the outcomes of patients undergoing ICI plus chemotherapy were scarce. A post hoc analysis of the IMpower 150 trial^44^ analyzed the association of the administration of PPIs with treatment response in patients with nonsquamous NSCLC receiving a combination of atezolizumab, bevacizumab, carboplatin, and paclitaxel, 1 of the ICI plus chemotherapy regimens approved in clinical practice. In that study,^44^ history of PPI use was not significantly associated with the PFS of patients receiving atezolizumab, bevacizumab, carboplatin, and paclitaxel therapy but was associated with a shorter OS. These observations indicated that the clinical association of PPI administration with therapeutic outcomes would be larger in patients with NSCLC with a PD-L1 TPS of 50% or more undergoing ICI monotherapy than in those receiving ICI plus chemotherapy regimens. However, the clinical association of a history of PPI use with ICI plus chemotherapy regimens remains unclear, and further investigations are needed to reveal the underlying mechanisms and their contributing factors.

The present study indicated that a history of PPI use, but not a history of systemic antimicrobial therapy, was associated with treatment response in the pembrolizumab monotherapy group. There are several possible reasons for this difference in the clinical impact of these drugs on the therapeutic outcomes of ICI monotherapy. First, in this study, a history of antibiotics was defined as a history of administration within 30 days before the start of the first chemotherapy treatment, whereas a history of PPI use was defined as a history of administration of PPIs at first-line treatment initiation. Thus, the administration of PPIs persisted during the first-line treatment and may have had a prolonged impact on the therapeutic outcomes of ICIs compared with antibiotics. Second, the magnitude of the effect size on the gut microbiota may differ for these drugs. In a Japanese prospective study^45^ that investigated the association of medication with the gut microbiome, medications for the digestive tract were more associated with changes in the gut microbiome structure than other categories of drugs, including systemic antibiotics, and PPIs and osmotic laxatives were significantly associated with changes in the gut microbiome. Notably, that study^45^ also indicated that a markedly altered microbiome has the resilience to return to its baseline state after cessation of the causative drug. Taken together, among the concomitant drugs investigated, PPIs appear to have the most negative associations with therapeutic outcomes of ICI monotherapy, but the withdrawal of PPIs may reverse the therapeutic response. Nevertheless, further observational studies are needed to test this hypothesis.

The present study focused on concomitant drugs as clinical factors associated with poorer treatment outcomes of ICI monotherapy, providing a useful clinical biomarker in choosing ICI with or without chemotherapy for patients with advanced NSCLC with a high PD-L1 TPS. Several previous studies^16,17,18^ showed no difference in OS for ICI plus chemotherapy over ICI monotherapy in patients with NSCLC with high PD-L1 expression. However, a previous study^18^ showed ICI plus chemotherapy was associated with survival benefits during the first 6 months in patients with advanced NSCLC with a PD-L1 TPS of 50% or more and also during the first 12 months in those with a PD-L1 TPS of 90% or more compared with ICI monotherapy, which suggested that patients with a higher risk of early mortality or progression with ICI monotherapy may be better suited for ICI plus chemotherapy than ICI monotherapy. In addition, regarding OS, the number of events in our study was small, and the contribution of ICI plus chemotherapy to long-term prognosis over ICI monotherapy in patients with a history of PPI use is still unknown. Considering these findings, further studies with long-term follow-up are needed to clarify these associations.

Our study highlighted an important clinical issue regarding assessing the appropriateness of PPI prescriptions, particularly in patients with NSCLC for whom the use of ICI plus chemotherapy is challenging. Although PPIs are among the most widely used medications globally and demonstrate positive outcomes for treating and preventing acid-related disorders, they have the potential to cause severe adverse effects with prolonged use, such as an increased risk of enteric *Clostridium difficile* infection, osteoporosis, and metabolic disorders.^46,47,48^ Despite some guidelines^48,49,50^ suggesting discontinuing PPI use after 4 to 8 weeks or regular reassessment if symptoms persist, PPIs are being prescribed off-label, at higher than recommended doses, for longer durations than recommended, and without an identified indication. For patients with NSCLC with high PD-L1 TPS, pembrolizumab monotherapy and ICI plus chemotherapy have been established as standard first-line therapies. However, whether ICI plus chemotherapy is effective and safe for frail patients, such as older patients or those with poor ECOG PS, remains uncertain.^7,8,10,11,12^ In contrast, several previous studies^51,52^ have shown the efficacy and safety of pembrolizumab monotherapy in these frail populations; therefore, pembrolizumab monotherapy may be a more reasonable treatment option than ICI plus chemotherapy for frail patients with NSCLC with high PD-L1 TPS in a clinical setting. Considering the treatment strategy for these clinical patients, we should recognize that PPI use may be negatively associated with the treatment outcome of ICI monotherapy. The necessity of administering PPIs should be assessed before initiating first-line cancer treatment.

### Limitations

The present study had some limitations. First, this study had a multicenter retrospective design. Therefore, the possibility of selection bias could not be ruled out. However, the patients were consecutively enrolled, and PSM was conducted to reduce selection bias. Nevertheless, as the treatment decision was based on the clinical fitness of each patient, the possibility of selection bias cannot be completely ruled out. Second, because the study is retrospective, the evaluation schedule of treatment outcomes was unplanned. Therefore, PFS as the primary end point of this study may be unreliable compared with prospective studies. Additionally, the clinical data of concomitant medication were retrospectively collected from the electronic medical records of the patients, which may not be as precise as they would be if they were obtained directly from a clinical trial. Third, since PSM was used to reduce patient background bias, a smaller sample size than the overall population was inevitable. Specifically, the patient group with a history of PPI use represented approximately 30% of the overall population. Fourth, the study included only Japanese patients, and previous studies have elucidated substantial divergences in the gut microbiome structure between healthy individuals of different races and ethnicities.^53^ This diversity may preclude the generalizability of our findings to patients from other countries. On the basis of these limitations, although the results of our study are clinically important, they may not be conclusive and generalizable. Thus, confirmation with larger global cohort is required to validate our findings.

## Conclusions

In this cohort study, a history of PPI use was independently associated with worse treatment outcomes in patients with NSCLC with a PD-L1 TPS of 50% or more who were treated with pembrolizumab monotherapy but not those treated with ICI plus chemotherapy. PPI use may thus be an important clinical factor that should be considered in choosing treatment with ICI with or without chemotherapy in this population. Furthermore, when considering treatment strategies for patients in whom ICI plus chemotherapy is challenging, the appropriateness and necessity of PPI prescriptions should be assessed before initiating first-line treatment.

## Notes

Supplement 1. **eTable 1.** Patient Characteristics in All Patients (N=425) **eFigure 1.** Treatment Outcomes in All Patients (N=425) **eFigure 2.** Objective Response Rate and Disease Control Rate. Adjusted by Propensity Score Matching in All Patients (N=262) **eTable 2.** Patient Characteristics Adjusted by Propensity Score Matching in Patients With PPI Administration History (N=68) **eTable 3.** Patient Characteristics Adjusted by Propensity Score Matching in Patients With PPI Administration History (N=188) **eTable 4.** Logistic Regression Analysis for Factors Associated With Administration of PPI at Baseline (N=425)

Supplement 2.
Data Sharing Statement
