# Supplementary material for: Concomitant Proton Pump Inhibitor Use With Pembrolizumab Monotherapy vs Immune Checkpoint Inhibitor Plus Chemotherapy in Patients With Non−Small Cell Lung Cancer
Source: JAMA Netw Open. 2023 Jul 11;6(7):e2322915. doi: 10.1001/jamanetworkopen.2023.22915 (PMC10336622; doi:10.1001/jamanetworkopen.2023.22915)
Supplement: Supplement 1. — eTable 1. Patient Characteristics in All Patients (N=425) eFigure 1. Treatment Outcomes in All Patients (N=425) eFigure 2. Objective Response Rate and Disease Control Rate. Adjusted by Propensity Score Matching in All Patients (N=262) eTable 2. Patient Characteristics Adjusted by Propensity Score Matching in Patients With PPI Administration History (N=68) eTable 3. Patient Characteristics Adjusted by Propensity Score Matching in Patients With PPI Administration History (N=188) eTable 4. Logistic Regression Analysis for Factors Associated With Administration of PPI at Baseline (N=425) [file jamanetwopen-e2322915-s001.pdf]

Supplemental Online Content

Kawachi H, Yamada T, Tamiya M, et al. Concomitant proton pump inhibitor use with pembrolizumab monotherapy vs immune checkpoint inhibitor plus chemotherapy in patients with non−small cell lung cancer. *JAMA Netw Open*. 2023;6(7):e2322915.
doi:10.1001/jamanetworkopen.2023.22915

eTable 1. Patient Characteristics in All Patients (N=425)
eFigure 1. Treatment Outcomes in All Patients (N=425)
eFigure 2. Objective Response Rate and Disease Control Rate. Adjusted by Propensity Score Matching in All Patients (N=262)
eTable 2. Patient Characteristics Adjusted by Propensity Score Matching in Patients With PPI Administration History (N=68)
eTable 3. Patient Characteristics Adjusted by Propensity Score Matching in Patients With PPI Administration History (N=188)
eTable 4. Logistic Regression Analysis for Factors Associated With Administration of PPI at Baseline (N=425)

This supplemental material has been provided by the authors to give readers additional information about their work.

© 2023 Kawachi H et al. *JAMA Network Open.*

eTable 1. Patient Characteristics in All Patients (N=425)

**Patient characteristics**	**Pembrolizumab group (N=271)**	**ICI plus Chemotherapy group (N=154)**	**P value**
Age (years)			
Median (range)	72 (43-90)	68.5 (36-86)	< .001
＜75 years	165 (61)	134 (87)	< .001
≧75 years	106 (39)	20 (13)	
Sex			
Male	215 (79)	121 (79)	.85
Female	56 (21)	33 (21)	
Smoking status			
Never-smoker	28 (10)	21 (14)	.31
Current or former smoker	243 (90)	133 (86)	
ECOG PS			
0-1	215 (79)	139 (90)	.004

© 2023 Kawachi H et al. *JAMA Network Open.*

2-4	56 (21)	15 (10)	
Histology			
Squamous cell carcinoma	80 (29)	40 (26)	.73
Adenocarcinoma	153 (56)	92 (60)	
Other	38 (14)	22 (14)	
PD-L1 status			
50-74%	104 (38)	51 (33)	.046
75-89%	63 (23)	53 (34)	
90-100%	104 (38)	50 (32)	
Stage			
IVA	94 (35)	48 (31)	.008
IVB	118 (44)	88 (57)	
Recurrence	59 (22)	18 (12)	
PPI			
Administered	95 (35)	39 (25)	.04
Antibiotics			

© 2023 Kawachi H et al. *JAMA Network Open.*

Administered	52 (19)	18 (12)	.045
Steroids			
Administered	14 (5)	7 (5)	.78
BMI			
< 20	80 (30)	48 (31)	.72
≧20	191 (70)	106 (69)	
Liver metastasis	35 (13)	23 (15)	.56
Brain metastasis	51 (19)	28 (18)	.87
Treatment regimen			
Pembrolizumab	271 (100)		
CBDCA/PTX/Pembrolizumab		1 (1)	
CBDCA/nab-PTX/Pembrolizumab		51 (33)	
CBDCA/PEM/Pembrolizumab		43 (28)	
CDDP/PEM/Pembrolizumab		28 (18)	
CBDCA/PEM/Atezolizumab		1 (1)	

© 2023 Kawachi H et al. *JAMA Network Open.*

CBDCA/PTX/Atezolizumab	1 (1)
CBDCA/PTX/BEV/Atezolizumab	18 (12)
CBDCA/nab-PTX/Atezolizumab	11 (7)

ICI, immune checkpoint inhibitor; ECOG PS, Eastern Cooperative Oncology Group Performance Status; PD-L1, programmed death ligand 1; PPI, proton pump inhibitor; BMI, body mass index; CBDCA, carboplatin; CDDP, cisplatin; PEM, pemetrexed; nab-PTX, nanoparticle albumin-bound paclitaxel; PTX, paclitaxel; BEV, bevacizumab.

© 2023 Kawachi H et al. *JAMA Network Open.*

eFigure 1 Treatment Outcomes in All Patients. (A) ORR and DCR. (B, C) Kaplan–Meier survival curves showing the progression-free survival (B) and overall survival (C) (N = 425). ICI/Chemo, immune checkpoint inhibitor plus chemotherapy; ORR, objective response rate; DCR, disease control rate.

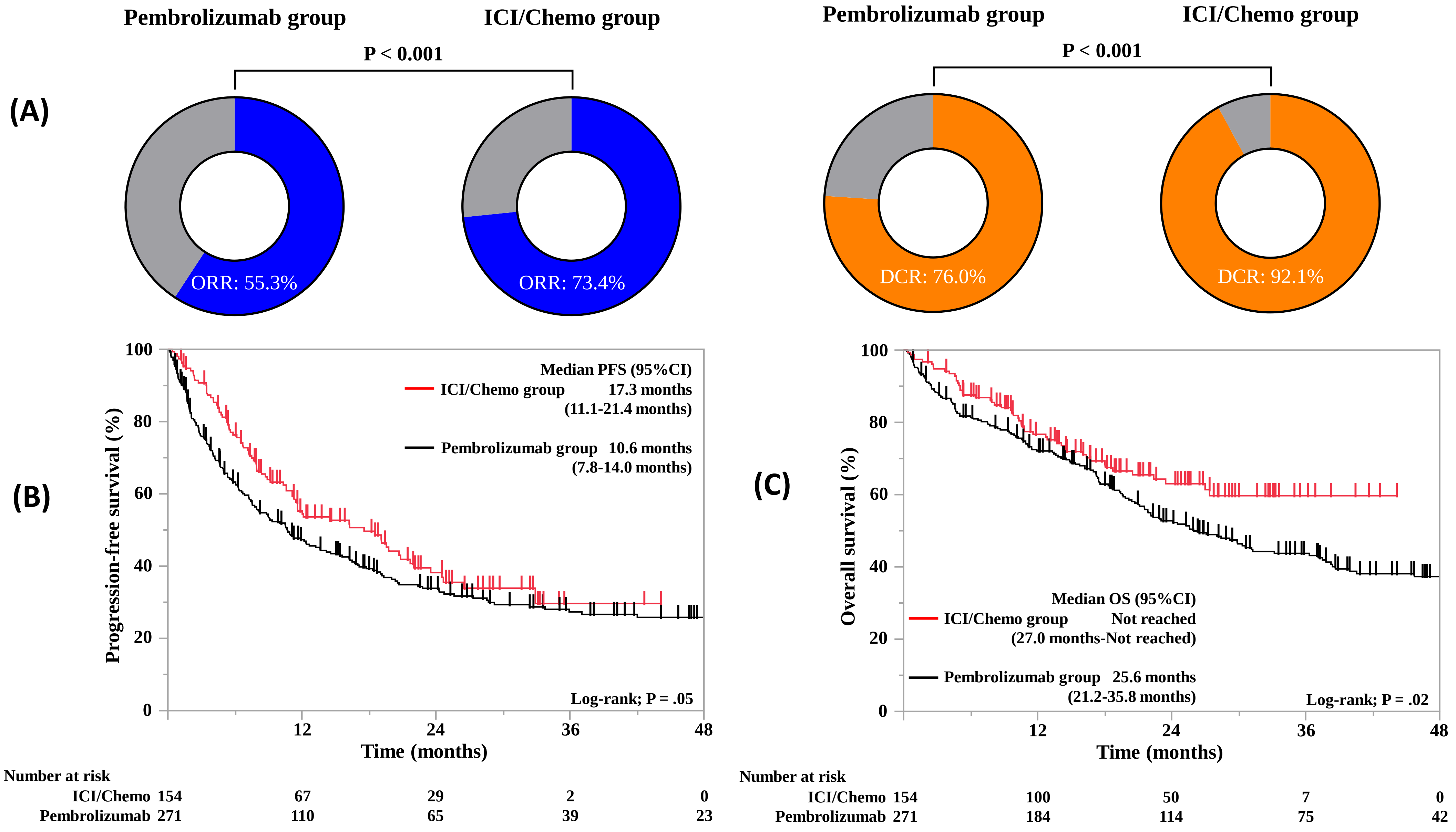

© 2023 Kawachi H et al. *JAMA Network Open.*

eFigure 2. Objective Response Rate and Disease Control Rate Adjusted by Propensity Score Matching in All Patients (N=262). ICI/Chemo, immune checkpoint inhibitor plus chemotherapy; ORR, objective response rate; DCR, disease control rate.

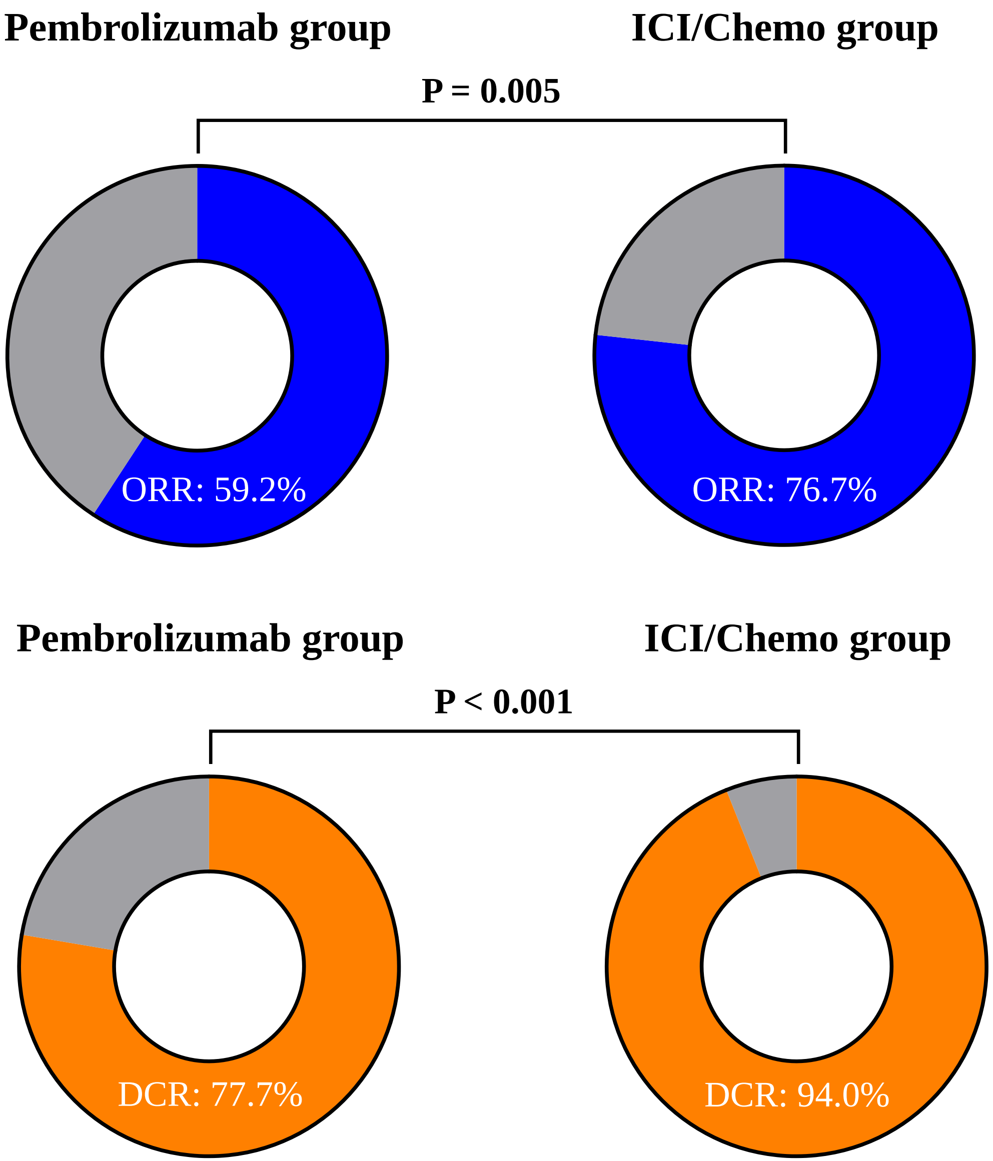

© 2023 Kawachi H et al. *JAMA Network Open.*

eTable 2. Patient Characteristics Adjusted by Propensity Score Matching in Patients with PPI Administration History (N=68)

**Patient characteristics**	**Pembrolizumab group (N=34)**	**ICI plus Chemotherapy group (N=34)**	**P value**
Age (years)			
Median (range)	72 (45-82)	69.5 (48-80)	.17
＜75 years	26 (76)	26 (76)	> .99
≧75 years	8 (24)	8 (24)	
Sex			
Male	30 (88)	28 (82)	.49
Female	4 (12)	6 (18)	
Smoking status			
Never-smoker	1 (3)	2 (6)	.56
Current or former smoker	33 (97)	32 (94)	
ECOG PS			
0-1	29 (85)	29 (85)	> .99

© 2023 Kawachi H et al. *JAMA Network Open.*

2-4	5 (15)	5 (15)	
Histology			
Squamous cell carcinoma	7 (21)	9 (26)	.76
Adenocarcinoma	22 (65)	19 (56)	
Other	5 (15)	6 (18)	
PD-L1 status			
50-74%	11 (32)	12 (35)	.95
75-89%	8 (24)	7 (21)	
90-100%	15 (44)	15 (44)	
Stage			
IVA	7 (21)	13 (38)	.24
IVB	20 (59)	17 (50)	
Recurrence	7 (21)	4 (12)	
Antibiotics			
Administered	8 (24)	6 (18)	.55
Steroids			

© 2023 Kawachi H et al. *JAMA Network Open.*

Administered	4 (12)	5 (15)	.68
BMI			
< 20	8 (24)	11 (32)	.42
≧20	26 (76)	23 (68)	
Liver metastasis	1 (3)	2 (6)	.56
Brain metastasis	7 (21)	5 (15)	.53
Treatment regimen			
Pembrolizumab	34 (100)		
CBDCA/nab-PTX/Pembrolizumab		12 (35)	
CBDCA/PEM/Pembrolizumab		7 (21)	
CDDP/PEM/Pembrolizumab		7 (21)	
		5 (15)	
CBDCA/PTX/BEV/Atezolizumab			
CBDCA/nab-PTX/		3 (9)	

© 2023 Kawachi H et al. *JAMA Network Open.*

Atezolizumab

PPI, proton pump inhibitor; ICI, immune checkpoint inhibitor; ECOG PS, Eastern Cooperative Oncology Group Performance Status; PD-L1, programmed death ligand 1; BMI, body mass index; CBDCA, carboplatin; CDDP, cisplatin; PEM, pemetrexed; nab-PTX, nanoparticle albumin-bound paclitaxel; PTX, paclitaxel; BEV, bevacizumab.

© 2023 Kawachi H et al. *JAMA Network Open.*

eTable 3. Patient Characteristics Adjusted by Propensity Score Matching in Patients With PPI Administration History (N=188)

**Patient characteristics**	**Pembrolizumab group (N=95)**	**ICI plus Chemotherapy group (N=95)**	**P value**
Age (years)			
Median (range)	68 (43-85)	68 (36-86)	.56
＜75 years	82 (86)	83 (87)	.83
≧75 years	13 (14)	12 (13)	
Sex			
Male	21 (22)	18 (19)	.59
Female	74 (78)	77 (81)	
Smoking status			
Never-smoker	13 (14)	13 (14)	> .99
Current or former smoker	82 (86)	82 (86)	
ECOG PS			
0-1	86 (91)	85 (89)	.81

© 2023 Kawachi H et al. *JAMA Network Open.*

2-4	9 (9)	10 (11)	
Histology			
Squamous cell carcinoma	22 (23)	27 (28)	.68
Adenocarcinoma	62 (65)	59 (62)	
Other	11 (12)	9 (9)	
PD-L1 status			
50-74%	36 (38)	30 (32)	.65
75-89%	47 (49)	51 (54)	
90-100%	12 (13)	14 (15)	
Stage			
IVA	37 (39)	32 (34)	.68
IVB	46 (49)	48 (51)	
Recurrence	11 (12)	14 (15)	
Antibiotics			
Administered	16 (17)	9 (9)	.13
Steroids			

© 2023 Kawachi H et al. *JAMA Network Open.*

Administered	2 (2)	4 (4)	.41
BMI			
< 20	31 (33)	30 (32)	.88
≧20	64 (67)	65 (68)	
Liver metastasis	14 (15)	17 (18)	.56
Brain metastasis	21 (22)	19 (20)	.72
Treatment regimen			
Pembrolizumab	95 (100)		
CBDCA/PTX/Pembrolizumab		1 (1)	
CBDCA/nab-PTX/Pembrolizumab		31 (33)	
CBDCA/PEM/Pembrolizumab		27 (28)	
CDDP/PEM/Pembrolizumab		16 (17)	
CBDCA/PEM/Atezolizumab		1 (1)	
CBDCA/PTX/Atezolizumab		1 (1)	
		12 (13)	

© 2023 Kawachi H et al. *JAMA Network Open.*

CBDCA/PTX/BEV/Atezolizumab	
CBDCA/nab-PTX/Atezolizumab	7 (7)

PPI, proton pump inhibitor; ICI, immune checkpoint inhibitor; ECOG PS, Eastern Cooperative Oncology Group Performance Status; PD-L1, programmed death ligand 1; BMI, body mass index; CBDCA, carboplatin; CDDP, cisplatin; PEM, pemetrexed; nab-PTX, nanoparticle albumin-bound paclitaxel; PTX, paclitaxel; BEV, bevacizumab.

© 2023 Kawachi H et al. *JAMA Network Open.*

eTable 4. Logistic Regression Analysis for Factors Associated With Administration of PPI at Baseline (N=425)

Characteristics	Univariate		Multivariate	
	OR (95% CI)	P-value	OR (95% CI)	P-value
Age				
≥75 years (vs.<75 years)	1.77 (1.14-2.74)	.01	1.73 (1.10-2.75)	.02
Sex				
female (vs. male)	1.00 (0.60-1.65)	.99	1.54 (0.83-2.82)	.17
Smoking status				
current or former smoker (vs. never)	2.21 (1.04-4.71)	.04	2.72 (1.11-6.67)	.03
ECOG PS				
2-4 (vs. 0-1)	2.03 (1.20-3.41)	.008	1.56 (0.87-2.79)	.14
Histology				

© 2023 Kawachi H et al. *JAMA Network Open.*

squamous (vs. non-squamous)	0.91 (0.57-1.43)	.67	1.22 (0.74-2.00)	.43
PD-L1 status				
50-89% (vs.≧90%)	0.74 (0.49-1.13)	.16	1.17 (0.74-1.84)	.50
Stage				
IVA or IVB (vs. Recurrence)	1.02 (0.60-1.74)	.94	0.84 (0.48-1.49)	.56
Antibiotics				
yes (vs. no)	2.59 (1.53-4.36)	<.001	2.40 (1.37-4.21)	.002
Steroids				
yes (vs. no)	3.80 (1.54-9.41)	.004	3.86 (1.37-10.93)	.01
BMI				
< 20 (vs.≧20)	0.88 (0.56-	.59	0.76 (0.46-	.27

© 2023 Kawachi H et al. *JAMA Network Open.*

	1.39)		1.24)	
Liver metastasis				
yes (vs. no)	0.72 (0.39-1.36)	.32	0.65 (0.33-1.26)	.20
Brain metastasis				
yes (vs. no)	1.00 (0.60-1.70)	.98	0.84 (0.48-1.49)	.56

PPI, proton pump inhibitor; ECOG PS, Eastern Cooperative Oncology Group Performance Status; PD-L1, programmed death ligand 1; BMI, body mass index; OR, odds ratio; CI, confidence interval.

© 2023 Kawachi H et al. *JAMA Network Open.*
