# Supplementary material for: Concomitant Proton Pump Inhibitor Use With Pembrolizumab Monotherapy vs Immune Checkpoint Inhibitor Plus Chemotherapy in Patients With Non−Small Cell Lung Cancer
Source: JAMA Netw Open. 2023 Jul 11;6(7):e2322915. doi: 10.1001/jamanetworkopen.2023.22915 (PMC10336622; doi:10.1001/jamanetworkopen.2023.22915)
Supplement: Supplement 2. — Data Sharing Statement [file jamanetwopen-e2322915-s002.pdf]

Data Sharing Statement

Kawachi. Concomitant Proton Pump Inhibitor Use With Pembrolizumab Monotherapy vs Immune Checkpoint Inhibitor Plus Chemotherapy in Patients With Non−Small Cell Lung Cancer. *JAMA Netw Open*. Published July 11, 2023.
doi:10.1001/jamanetworkopen.2023.22915

Data
Data available: No
